# Research and Remedies

**Published:** 1913-08-23

**Authors:** 


### RESEARCH AND REMEDIES.

### The Treatment of Diphtheria Carriers.

In several infective diseases it is found that certain individuals continue to harbour the micro-organisms of the malady long after all symptoms have disappeared, and these "carriers" are often extremely refractory to all treatment. In the case of diphtheria, Schiotz, a Danish physician, noticed four years ago a kind of antagonism between the staphylococcus and the diphtheria bacillus, and the idea occurred to him of utilising this property in attempting to cure diphtheria carriers. He accordingly inoculated six diphtheria carriers with staphylococci from the throat of a patient in a surgical ward, and all six shortly ceased to harbour diphtheria bacilli. Page of Manila carried the method a step further by using cultures of staphylococci as a throat spray for this purpose, and several American physicians have reported favourably on it. On the Continent and in this country the idea seems not to have been taken up at all until Dr. J. D. Rolleston tried it lately; he treated ten chronic carriers by spraying and swabbing the nose and throat with a bouillon culture of *Staphylococcus pyogenes aureus*. In six faucial cases the findings became negative (*i.e.*, the carriers ceased to carry) within a week. In two nasal cases the treatment was ineffective. A mild form of sore throat is produced by the treatment, but no complications ensued.

### The Treatment of Tetanus.

The antitoxin treatment of tetanus having now been thoroughly tested over a considerable series of years and having disappointed expectations, attention has lately been redirected to a form of treatment originated years ago by Baccelli. This consists of subcutaneous injections of 3 per cent. carbolic acid, given frequently enough to make the total quantity of pure phenol equal to 5 to 7½ grains per day. In the *Medical Record* some recent statistics of the result of this method are given. Imperiali thus treated one hundred and thirty-eight cases of tetanus, among which there ensued one hundred and fifteen recoveries and twenty-three deaths. In a later paper Baccelli himself published one hundred and ninety cases with a mortality of 17.36 per cent. Goulyæff, himself at one time a keen supporter of the serum treatment, gave Baccelli's method a trial in two apparently very severe cases, and recovery followed in both of them. The method is at any rate very simple, and in view of the results claimed in Italy should certainly be tried.

